# Recent trends in an uncommon method of carbon monoxide suicide

**DOI:** 10.1007/s12024-024-00810-x

**Published:** 2024-04-11

**Authors:** Lilli Stephenson, Corinna Van Den Heuvel, Melissa Humphries, Roger W. Byard

**Affiliations:** 1 School of Biomedicine, The University of Adelaide, Adelaide, SA 5000 Australia; 2 School of Computer and Mathematical Sciences, The University of Adelaide, Adelaide, SA 5000 Australia; 3 Forensic Science SA (FSSA), 21 Divett Pl, Adelaide, SA 5000 Australia; 4 School of Biomedicine Level 2 Helen Mayo North , The University of Adelaide, Frome Road, Adelaide, SA 5005 Australia

**Keywords:** Suicide, Charcoal burning, Carbon monoxide, Vehicle exhaust

## Abstract

**Purpose:**

The most prevalent method of carbon monoxide (CO) suicide is inhalation of vehicle exhaust (VE). However, a new method of CO suicide has recently emerged involving charcoal burning (CB) in a confined space to produce fatal CO levels. This method has been reported from countries in Asia, associated with economic instability and media reporting of high-profile celebrity cases. The current study was undertaken to analyze rates and characteristics of CB suicides in South Australia (SA) for comparison with respect to their characteristics and scene, autopsy and toxicology findings.

**Methods:**

A search was undertaken for all intentional fatal carbon monoxide poisonings in SA between 2000 and 2019. Collected variables included age, sex, cause of death, location of death, decedent histories, scene, autopsy and toxicology findings and manner of death. Statistical analyses were performed using R (version 4.2.3).

**Results:**

There was a significant decrease in VE suicides (*p* < 0.05) and a significant increase in CB suicides (*p* < 0.001) over the 20-year period. Those who used CB were found to be, on average, between 1.5 and 15.8 years younger than those who used VE (*p* = 0.017). The risk factors for CB suicide included psychological/psychiatric conditions and financial problems, while VE suicides were associated with a history of physical problems and contact with the legal system. External and internal autopsy findings were consistent with the literature.

**Conclusion:**

CB suicide is perceived to be widely accessible and painless and is therefore becoming a popular suicide method. Monitoring future trends will be important to determine whether intervention is required.

**Supplementary Information:**

The online version contains supplementary material available at 10.1007/s12024-024-00810-x.

## Introduction

Carbon monoxide (CO) is an odorless, colorless gas that may cause death by displacing circulating oxygen or by direct cellular toxicity. Deaths may be due to accidents, suicides, or homicides [1, 2]. Suicide by CO poisoning is most commonly achieved by inhaling exhaust fumes from a running vehicle in a confined space. The demographic and spatial trends of cases utilizing this method are well documented [3]. However, in certain cases barbeque charcoal is used to produce fatal CO levels. This method has been reported mostly in East/Southeast Asia with differing prevalence and demographic trends between countries [4]. In Hong Kong, the method emerged in the late 1990’s [5, 6] after a case of charcoal-burning (CB) suicide was presented with visual images in the news in November 1998, with nine more CB suicides occurring in the following month [7]. This was also thought to be influenced by the economic downturn and increased unemployment rates in Hong Kong at the time [8]. In Taiwan, the number of CB suicides gradually increased from the late 1990’s [6, 9, 10]. The use of this method was delayed in Korea, but its popularization was driven by media reporting of a high-profile Korean celebrity case in 2008 [11]. This event is recognized as the catalyst for a trend of copycat suicides throughout the region, with CB suicides accounting for < 1% of suicides prior to the report, which increased to almost 5% in the 12 months after it was released [11, 12]. Utilization of this suicide method has also been observed in other countries around the world, albeit in lower numbers [13–21]. The following study was undertaken to analyze the rate and profile of CB suicides in South Australia (SA) compared to previous reports. In addition, the study also aims to compare the rate and characteristics of CB and vehicle exhaust (VE) suicides in the SA autopsy population.

## Materials and methods

The Toxicology Database at Forensic Science SA (FSSA) was searched for all post-mortem CO detections over a 20-year period from 1 January 2000 to 31 December 2019 in SA. Relevant cases were then cross-referenced against autopsy reports to identify deaths attributed to intentional (i.e., suicidal) CO poisoning. Additional information was retrieved from autopsy reports, including age, sex, cause of death, location of death, decedent histories, scene findings, autopsy and toxicology findings and manner of death. Age was categorized into the following groups: young person (15–24 years), adult (25–64 years) and elderly person (65 + years).

**Fig. 1:**
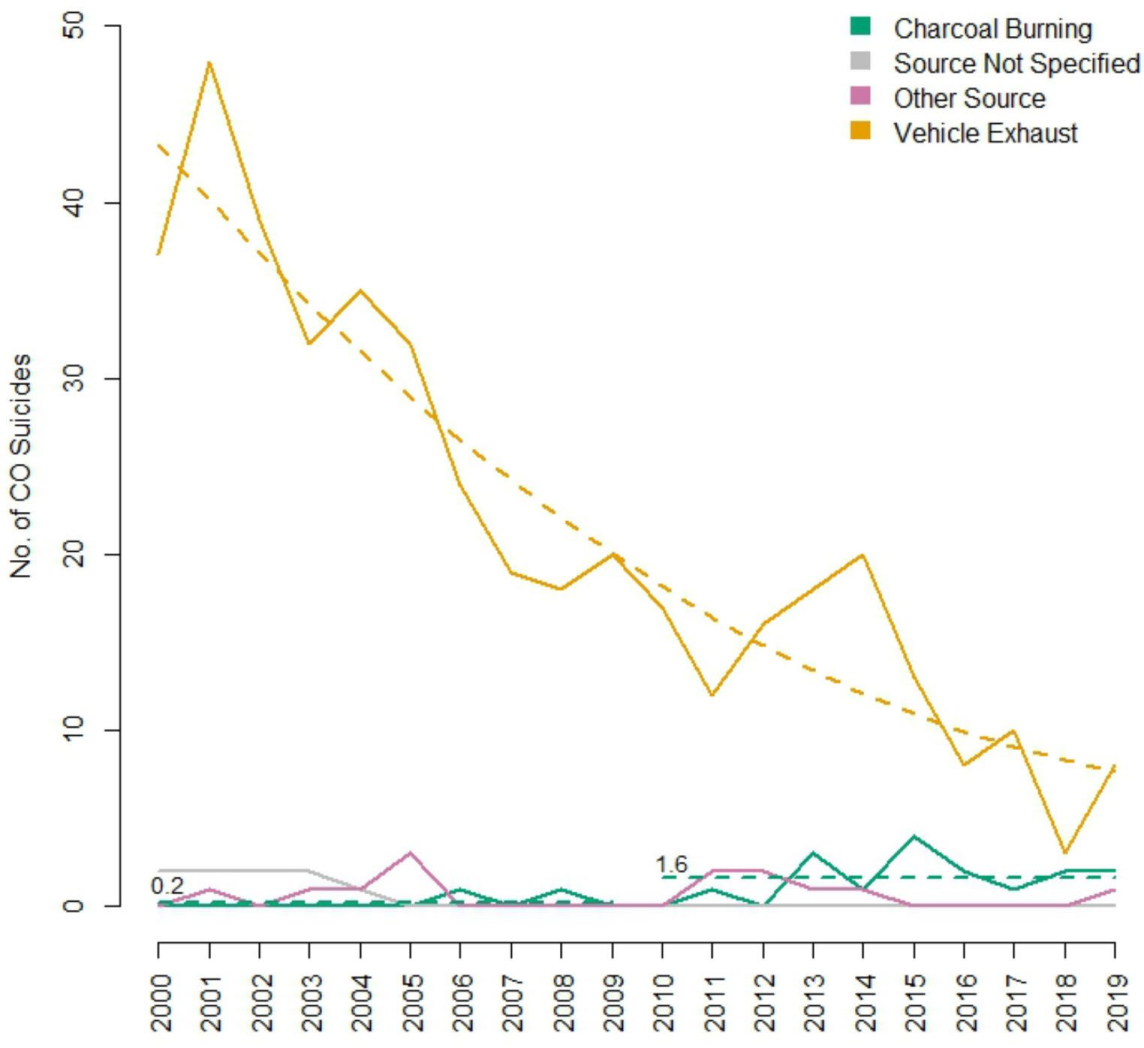
Number of CO suicides by method in SA between 2000–2019 with a fitted quadratic regression for VE suicides and two 10-year averages for CB suicides

Statistical analyses were performed using R (version 4.2.3) [22]. A polynomial regression characterized trends in VE suicides. A Poisson regression characterized trends in CB suicides. Data pertaining to CB suicides shown in Fig. 1 is presented as the average number of deaths across two 10-year periods (2000–2009, 2010–2019) indicated by the horizontal dashed green lines.

Ethics approval for the data used in this study was granted by the University of Adelaide Human Research Ethics Committee (H-2020-033).

Proximal risk factors for intentional self-harm were based on the International Classification of External Causes of Injuries (ICECI) [23].

Data for all suicides in SA was sourced from the Australian Institute of Health and Welfare [24].

A review of the literature was undertaken through PubMed to identify articles which present longitudinal, retrospective reviews of CO and/or CB suicides within a population(s). Search words used were ‘carbon monoxide’, ‘charcoal’ and ‘suicide’. In total, 90 papers were reviewed, of which 20 were included in the literature review. Only papers written in English and that provided adequate details of the study period, proportions and/or demographic characteristics (where available) were included.

## Results

### Literature review

As expected, case reports from countries in Asia (e.g. Taiwan, Hong Kong, China) demonstrated a markedly higher number of CB suicides as a proportion of total suicides compared to most other countries (Appendix A, Table A.1). In contrast, countries throughout Europe and North America reported significantly lower proportions, ranging from < 1–6% and was highest in the United States (US).

The mean age of decedents was approximately 45 years, and the largest proportion of decedents were male (75–87%). Unfortunately, very few articles reported on the ethnicity of the decedents. Most decedents from the US were “white” (88%) while most decedents from Canada were Asian (92.8%).

### Source of CO

Between 2000 and 2019, 4117 suicides occurred in SA. Over this period, 11.5% of all suicides were attributed to CO poisoning (*n* = 472). For specific CO suicide methods, VE and CB accounted for 10.6% (*n* = 438) and 0.4% (*n* = 18) of all suicides in SA respectively.

Among all cases of CO poisoning, 97.6% of VE deaths were suicides (438/449) and 90% of CB deaths were suicides (18/20). However, in cases where the source of CO was either unknown (*n* = 5) or “other” (*n* = 28), the proportion of cases that were suicides were 60% and 46.4% respectively (Fig. 1).

Overall, the leading sources of CO toxicity in CO-related suicides were inhalation of VE followed by CB, ‘other’ sources of CO toxicity (*n* = 13) and cases where the source of CO was unknown (*n* = 3). ‘Other’ sources of CO included inhaling exhaust from a fuel generator, products of combustion, a CO cylinder, and exhaust from a garden trimmer and collectively comprised less than 3% of all cases. Cases where the source of CO was unknown were confined to the first five years of the study period, influenced by less detailed reporting of scene findings during the earlier years.

While VE was the most common method of CO suicide compared to all other sources, there was a significant, quadratic decrease in the number of VE suicides over the study period indicated by the corresponding dashed line in Fig. 1 (see Appendix A, Table A.2 for full statistical analyses). This decrease was observed to be more rapid in the early years, which began to plateau toward a natural baseline as cases approached zero. The rate of change from year to year can be calculated using the equation:

$${\rm{Rate}}\,{\rm{of}}\,{\rm{change}}\,{\rm{ = }}\,{\rm{0}}{\rm{.142}}\, \times \,{\rm{Year}}\,{\rm{-}}\,{\rm{286}}{\rm{.4}}$$

For example, in 2002 the average number of deaths decreased by 2.12, compared to a decrease of 0.41 deaths in 2014. As illustrated in Fig. 1, the equation above suggests that VE suicides have consistently decreased over time in a parabolic shape, finally reaching a plateau towards the end of the study period. However, the equation should not be used as a prediction tool for future years (i.e. 2020 onwards) as it is unlikely that the trend will continue towards and reach zero but rather follow a more natural pattern of peaks and troughs as is reflected by the raw numbers.

While there were almost no CB suicides in the first 10 years of the study period (0.2 per year average), the rate of deaths increased significantly from 2010 onwards to an average of 1.6 suicides per year as indicated in Fig. 1 by the corresponding dashed lines (*p* < 0.001, see Appendix A, Table A.3 for full statistical analyses). Using the first 10-year period as a baseline, a Poisson distribution with rate $\lambda =0.2$, the probability of seeing 2, 3 or 4 deaths in any one year would be 0.017, which is extremely low. In the second 10-year period (2010–2019), 50% of the time case numbers of 2 or greater were observed, providing strong evidence of a significant increase in the rate of CB suicides.

### Demographic profile

Most decedents who used CB to commit suicide in SA were male and adults (78% and 72% respectively) (Table 1). For VE suicides, most decedents were also male (83%) and adults (82%). However, compared to CB suicides, there was a greater proportion of elderly persons in the VE cohort (12% compared to 6%). Conversely, a larger proportion of decedents who used CB were young persons (22% compared to 6%). A two-sample t-test showed that the mean age of decedents for CB and VE suicides (37.2 and 45.8 years respectively) was significantly different (*p* = 0.017), where CB decedents were, on average, between 1.5 and 15.8 years younger than VE decedents (see Appendix A, Table A.4 for full statistical analyses).

**Table 1:** Decedent characteristics and proximal risk factors for intentional self-harm for CB and VE suicides

	CB (*n* = 18)	VE (*n* = 438)
Decedent characteristics		
*Sex* Male Female	*n (%)* 14 (78) 4 (22)	*n (%)* 365 (83) 73 (17)
*Age category* Young person Adult Elderly person	*n (%)* 4 (22) 13 (72) 1 (6)	*n (%)* 28 (6) 358 (82) 52 (12)
*Ethnicity* Caucasian (“white”) Asian African Not stated	*n (%)* 8 (44) 2 (11) 1 (6) 7 (39)	*n (%)* 324 (74) 1 (0) 0 (0) 113 (26)
**Proximal risk factors for intentional self-harm**	*n (%)*	*n (%)*
Conflict in a romantic relationship	3 (17)	45 (10)
Death of a family member (or anniversary)	0 (0)	6 (1)
Physical problem HIV/AIDS Cancer Neurodegenerative condition Chronic pain Crohn’s disease Other	0 (0) 0 (0) 0 (0) 0 (0) 0 (0) 0 (0) 0 (0)	24 (5.5) 3 (1) 10 (2) 4 (1) 3 (1) 2 (0) 2 (0)
Psychological/psychiatric condition* Substance abuse Other (e.g. depression, schizophrenia)	13 (72) 2 (11) 11 (61)	189 (43) 33 (7) 176 (40)
Income Work-related Debt Unspecified	3 (17) 1 (6) 1 (6) 1 (6)	8 (2) 5 (1) 0 (0) 3 (1)
Abuse	0 (0)	2 (0)
Legal	0 (0)	13 (3)
Previous suicide attempt(s)	1 (6)	38 (9)
History of suicidal ideation	5 (28)	82 (19)
Suicide note	11 (61.1)	157 (35.8)

*Numbers do not total 100% as several decedents had both risk factors within this category

Decedents had varied racial profiles compared to studies from the US and Canada [21, 25]. For CB suicides, the majority of decedents in the current study were described as being of Caucasian appearance or “white” (*n* = 8, 44%). Less than five decedents were reported as being from Asian or African decent, with the ethnicity of the remaining decedents not stated. For VE suicides, most decedents were Caucasian or “white” (*n* = 324, 74%). Fewer than five decedents were Asian, and ethnicity was not stated in the remaining cases.

### Circumstances of death

An analysis of risk factors for CB and VE suicides revealed differences (Table 1). CB suicides were more closely associated with a history of a psychological or psychiatric condition and financial problems compared to VE suicides (72.2% vs. 43.2% and 16.7% vs. 1.8% respectively). However, for VE suicides, there was a larger proportion of cases where the decedent was suffering from some form of physical problem (e.g. cancer, neurodegenerative condition), or where the decedent had contact with the criminal justice system prior to death compared to CB suicides (5.5% vs. 0% and 2.9% vs. 0%).

While there were no cases of CB suicides involving more than one individual, seven cases of VE suicide involved two individuals in various contexts (i.e. murder-suicide, double-suicide etc.). There were several risk factors identified in the histories of the decedents in such cases including deteriorating health and contact with the legal justice system.

### Toxicology

The COHb level for CB suicides ranged from 37 to 85% (mean = 69.7%) and from 6 to 98% for VE suicides (mean = 74.4%), yet the difference was not significant (*p* = 0.25, see Appendix A, Table A.5 for full statistical analysis). However, it is worth noting that the average COHb level for CB suicides where decedents were found in a vehicle (*n* = 10) was 74.6% which was much closer to the mean COHb level in VE suicides.

Of the 18 CB suicides, only one case was associated with toxicity of other substances in conjunction with CO (i.e. mixed substance toxicity). This case involved the ingestion of several opioid, benzodiazepine and antihistamine sedatives (morphine, codeine, clonazepam, nitrazepam, chlorpheniramine) resulting in additive fatal respiratory depressant effects. The CO level in this case (83%) was not, however, significantly different to other cases.

Of the 438 VE suicides, only 15 (3.4%) were associated with mixed-substance toxicity (Table 2). The mean COHb level in cases attributed to mixed-substance toxicity was markedly lower compared to those attributed to CO toxicity alone (48% and 75.4% respectively). The substances co-implicated in cases of mixed-substance toxicity included various opioid, benzodiazepine and other sedatives with no demonstrable trends between cases. Cases with COHb levels of less than 30% were often seen in combination with significant cardiorespiratory disease (e.g. emphysema, lung carcinoma, coronary artery atherosclerosis) and/or a significantly elevated drug level(s).

**Table 2:** Toxicology findings for cases of mixed-substance toxicity in VE suicides

Case no.	COHb level (%)	Other substance(s)	Level (mg/L)	Cardiorespiratory disease
1	28	Propoxyphene	1.3	70% stenosis of coronary arteries
2	73	Zolpidem	0.93	None
3	81	Tramadol	3.5	None
4	14	Oxycodone	0.35	Extensive metastatic carcinoma of the lungs
5	89	Paracetamol Codeine	280 2.4	Atherosclerosis, anthracosis, emphysema
6	64	Olanzapine Codeine Diazepam Temazepam	0.78 1.1 0.1 0.4	Anthracosis, emphysema
7	16	Morphine Codeine Monoacetylmorphine	0.06 0.01 NQ	None
8	13	Temazepam	5.2	Atherosclerosis
9	70	Zolpidem	2.1	50% stenosis of coronary arteries
10	21	Doxylamine	0.5	Moderate coronary atheroma, anthracosis
11	53	Nitrazepam 7-aminonitrazepam Diazepam Nordiazepam Morphine	0.1 0.32 0.32 0.45 0.05	Mild coronary atheroma
12	85	Methadone Oxycodone Diazepam Nordiazepam Oxazepam Propoxyphene Codeine	0.56 0.74 0.4 0.14 0.15 0.11 0.13	50% stenosis of coronary arteries
13	20	Nitrazepam 7-aminonitrazepam Diazepam Nordiazepam Codeine	0.007 0.28 0.3 0.4 0.2	None
14	62	Codeine Morphine Paracetamol	1.2 0.016 130	None
15	31	Morphine Temazepam	0.22 0.12	Mild coronary atheroma, emphysema

NQ = not quantitated (“present”)

For both CB and VE suicides, there was no correlation between the presence of pre-existing cardiopulmonary disease and death at lower COHb levels. On average, there was only a 1.5% difference in the COHb level between decedents with both cardiovascular and respiratory disease and decedents without any disease.

### External examination

There were no significant external findings in cases of CB and VE suicide other than “cherry-red” lividity which was observed in most cases (> 89%, Table 3). Of note, this could be observed for a considerable time after death, up to 32 days for one case. For cases of VE suicide, soot or dirt staining was identified on the hands of 81 decedents (18%) and its absence was only documented in nine cases (2%). However, the presence or absence of soot staining on the hands was not reported in the largest proportion of cases (80%).

### Internal autopsy findings

Internal autopsy findings for CB and VE suicides were also consistent with the literature and are summarized in Table 3. For both methods, pulmonary congestion was consistently observed (> 81% of cases) except for those with marked putrefaction.

**Table 3:** External and internal autopsy findings for CB and VE suicides

	CB *n* = 18	VE *n* = 438
**Autopsy findings^**	*n (%)*	*n (%)*
*External soot staining on fingers/hands* Y N Not reported	0 (0) 0 (0) 18 (100)	81 (18) 9 (2) 348 (80)
*Pulmonary congestion and oedema* Y N Not reported Putrefaction	17 (94) 0 (0) 0 (0) 1 (6)	353 (81) 36 (8) 5 (1) 44 (10)
*Blood-stained pleural effusion* Y N Not reported	5 (28) 13 (72) 0 (0)	84 (19) 350 (80) 4 (1)
*Respiratory disease* Y Anthracosis Emphysema Other N Not reported Putrefaction	2 (11) 2 (11) 1 (6) 0 (0) 15 (83) 0 (0) 1 (6)	101 (23) 77 (18) 50 (11) 6 (1) 313 (71) 4 (1) 20 (5)
*Cardiovascular disease* Y Cardiomegaly Stenosis/atherosclerosis/atheroma N Not reported Putrefaction	5 (28) 2 (11) 4 (22) 13 (72) 0 (0) 0 (0)	170 (39) 6 (1) 169 (39) 260 (59) 4 (1) 4 (1)
*Cherry-red lividity of blood/internal organs* Y N Not reported Putrefaction	16 (89) 2 (11) 0 (0) 0 (0)	405 (92) 30 (7) 2 (1) 1 (0)
*Macroscopic brain pathology* Y Atheroma Congestion/swelling Uncal notching/cerebellar tonsillar grooving Dilatation of lateral & 3rd ventricles N Not reported Putrefaction	3 (17) 1 (6) 1 (6) 1 (6) 0 (0) 12 (66) 0 (0) 3 (17)	29 (7) 8 (2) 11 (2) 5 (1) 4 (1) 366 (83) 4 (1) 39 (9)
*Brain histology* Y Congestion Hypoxic ischemic encephalopathy Neuronal red/dark cell changes Microscopic subarachnoid hemorrhage N Not reported* Putrefaction	0 (0) 0 (0) 0 (0) 0 (0) 0 (0) 6 (34) 12 (66) 0 (0)	10 (2) 5 (1) 2 (1) 2 (1) 1 (0) 174 (40) 221 (50) 33 (8)

*Histology was not undertaken in several cases due to the cause of death being determined from the toxicology findings

^Unable to be assessed due to advanced decomposition (*n* = 3, *n* = 9)

### Scene findings

A suicide note was found in 61.1% and 35.8% of cases for CB and VE suicides respectively (Table 1). In all cases of CB suicide, the method used was indicated by burning (or burned) charcoal in some form of BBQ apparatus. More than 50% of CB decedents (*n* = 10) were found inside a vehicle. The remaining decedents were found in various locations in their homes (e.g. bedroom, bathroom). Conversely, for VE suicides the exhaust of a motor vehicle was diverted either to the interior of a vehicle or into a sealed room using piping.

## Discussion

The increasing prevalence of CB suicides, particularly in Asia, has been attributed to two main factors: economic hardship and publicizing of cases in the media. This has led to consideration of the risks of reporting of suicide methods and case-specific details in the media [26]. The decline in CO suicides utilizing VE has been widely reported and has often been attributed to the implementation of catalytic converters in vehicles. However, this causative relationship has not been substantiated in Australia. In 1995, suicide by VE was the second most common method of suicide second only to hanging, and had increased substantially despite the introduction of catalytic converters and CO exhaust limits in the 1980’s [3]. Issues which may affect the efficiency of catalytic converters with CO levels subsequently exceeding the legislated CO limit include engine idling from a cold start, with a 1–3 min delay in operating efficiency [3]. While the current study demonstrates a significant decrease in the number of VE suicides in SA from 2000 onwards, the reasons behind the decrease are unclear. Comparisons between non-pharmacological methods of suicide offer unique insights into the relationship between method accessibility and the evolution of suicide trends over time in response to legislative change and the implementation of preventative measures. It is likely that trends in preferred suicide methods are changing, particularly in response to the wide availability of suicide information online, as has been demonstrated in the use of inert gases such as helium or nitrogen [27, 28]. Other studies of the use of non-pharmacological substance-related suicide trends in SA by the authors have revealed a significant increase in the number of suicides using sodium nitrite, with a total of ten cases over the 20 year period which were most frequently observed among older males [29]. This trend is largely thought to be due to a recent increase in available literature online related to this specific method. Suicides using pesticides were found to be rare but consistent over time with 41 cases in total, and were similarly most prevalent amongst a middle-aged, male demographic [30]. While this suicide method has a long and prominent history driven by socioeconomic instability, it appears to be far less common in developed countries such as Australia. While neither of the latter methods are comparable in number to suicides involving CO from vehicle exhaust in SA, it is however similar to the number of CB suicides over the same time period. While trends in sodium nitrite suicides demonstrated a dramatic increase over time, the change in the number of CB suicides was much more subtle. Monitoring of trends associated with these substances moving forward will be important for the development of targeted suicide prevention strategies.

While there is a well-documented history of VE suicides among the Australian population, the incidence of CB suicides is a comparatively new phenomenon. Australia’s experience with CB burning fatalities began in the early 2000’s with a case of inadvertent CO exposure by burning charcoal in an enclosed space in Sydney [31]. While the subsequent implementation of mandatory CO warnings on all charcoal brickettes in Australia since 2009 has likely contributed to the near absence of accidental CB deaths in SA, interventions targeted at reducing the number of CB suicides may need to be considered. It appears that rates of CB suicide are increasing in western countries, which may also be contributed to by recent depictions of this method in popular culture including the viral television series’ ‘Squid Game’ [32] and ‘Beef’ [33], which aired on Netflix in 2021 and 2023 respectively. It will be important to monitor future trends to determine whether these additional media representations, or other factors, contribute to the increased prevalence of this method in western countries.

The demographic profile and proportions of CB suicide in the current study were similar to those reported in England and Wales [18], comprising less than 1% of all suicides over the 20-year period and comprised largely of Caucasian (“white”) decedents. While decedents for both CB and VE suicides were mostly male and adults, those who utilized VE were significantly older. In addition to these differences, the risk factors between CB and VE suicides were different. CB suicides showed a greater association with psychological/psychiatric conditions and financial problems, while VE suicides were more closely associated with a history of a physical problem (e.g. cancer or neurodegeneration) and contact with the criminal justice system.

The use of CB has also been reported in maternal-filicide-suicide cases [34, 35], with other examples of multiple-fatality CB suicides occurring [15, 36, 37]. However, while no multiple-fatality events were observed among the CB suicide cohort in the current study, several were observed for VE suicides. However, none were associated with filicide, but rather suicide-pacts.

COHb concentrations in excess of 60% are generally considered fatal [38], with severe poisoning occurring at concentrations greater than 30% [39]. In cases where the COHb level was less than 60%, this was largely explained by severe putrefaction and prolonged post-mortem intervals. Although well documented in the literature for a synergistic effect [40], the presence of pre-existing cardiopulmonary disease did not appear to influence the level of COHb required to produce a fatal outcome in the current series. However, for VE suicides associated with mixed-substance toxicity, COHb levels were lower among decedents who demonstrated cardiopulmonary disease, in addition to toxic or fatal drug levels. This is to be expected due to the additive depressant effects of sedative medications exacerbating the physiological impairment associated with CO intoxication.

It is thought that the characteristic sign of cherry-red lividity in cases of fatal CO exposure are observed more frequently with COHb concentrations in excess of 30% [41]. However, the current study demonstrated that putrefaction was the greatest determinant of whether this sign would be observed or not. Non-specific pulmonary oedema and organ congestion were also frequently observed [41]. There is disagreement in the literature as to whether necrosis of the globus pallidus should be considered a characteristic sign of CO poisoning. While it has been demonstrated in patients who have attempted and survived a CB suicide attempt [42], it does not appear to be evident in the autopsy population of the current study. A recent review of this specific neuropathology showed no association with fatal CO poisoning relative to other potential causes (drug overdose, hypertensive and/or atherosclerotic cardiovascular disease etc.) [43]. The results of this study agree with this premise, where no specific neuropathological findings were observed specifically related to CO poisoning.

In cases of CO suicide, the external autopsy and scene findings are often the two most important information sources in guiding investigations. In cases of VE suicide, efforts to divert exhaust to a confined area using piping, tape and often bedding are an obvious clue as to intent. The presence of soot on the hands is also a useful indicator that the decedent has set up the lethal system, when documented.

The presence of burned (or burning) coals in cases of an unexpected death, often within a BBQ apparatus, should also raise suspicions about the likelihood of suicide. For both CB and VE suicides, evidence of modifications such as tape, sheets or other materials to seal gaps (e.g. car windows, door frames etc.) are significant [44].

Unfortunately, as with other sources of non-pharmaceutical suicide (e.g., sodium nitrite) [29], charcoal is another material that is very difficult to regulate due to its widespread use and availability. CB suicide is also perceived to be accessible and painless and is therefore becoming a popular suicide method [45, 46]. The introduction of “deterrents” such as restricting access to retail BBQ charcoal have been proposed and implemented in Hong Kong and Taipei with mixed results [47, 48]. However, such interventions are also associated with potential problems in restricting BBQ charcoal sales and in customer profiling.

## Key points

1. Carbon monoxide suicides using vehicle exhaust significantly decreased over the 20-year study period
2. Use of charcoal burning is an emerging alternative method of carbon monoxide suicide
3. Decedents of charcoal burning suicide were up to 15.8 years younger than those who used vehicle exhaust
4. The psychological, social and physical risk factors for vehicle exhaust and charcoal burning suicide were notably different

## Electronic supplementary material

Below is the link to the electronic supplementary material.

Supplementary Material 1

## Data Availability

Not applicable.

## References

[1] Byard RW. Carbon monoxide - the silent killer. Forensic Sci Med Pathol. 2019;15(1):1–2. 10.1007/s12024-018-0040-5. 30390280 10.1007/s12024-018-0040-5

[2] Heath K, Byard RW. Lethal carbon monoxide toxicity in a concrete shower unit. Forensic Sci Med Pathol. 2019;15(1):133–5. 10.1007/s12024-018-9990-x. 29796749 10.1007/s12024-018-9990-x

[3] Routley VH, Ozanne-Smith J. The impact of catalytic converters on motor vehicle exhaust gas suicides. Med J Aust. 1998;168(2):65–7. 10.5694/j.1326-5377.1998.tb126713.x. 9469185 10.5694/j.1326-5377.1998.tb126713.x

[4] Chang SS, Chen YY, Yip PS, Lee WJ, Hagihara A, Gunnell D. Regional changes in charcoal-burning suicide rates in East/Southeast Asia from 1995 to 2011: a time trend analysis. PLoS Med. 2014;11(4):e1001622. 10.1371/journal.pmed.1001622. 24691071 10.1371/journal.pmed.1001622 PMC3972087

[5] Chang YH, Hsu CY, Cheng Q, Chang SS, Yip P. The evolution of the characteristics of charcoal-burning suicide in Hong Kong, 2002–2013. J Affect Disord. 2019;257:390–5. 10.1016/j.jad.2019.07.041. 31306989 10.1016/j.jad.2019.07.041

[6] Chen YY, Yip PS, Lee CK, Gunnell D, Wu KC. The diffusion of a new method of suicide: charcoal-burning suicide in Hong Kong and Taiwan. Soc Psychiatry Psychiatr Epidemiol. 2015;50(2):227–36. 10.1007/s00127-014-0910-4. 24912402 10.1007/s00127-014-0910-4

[7] Lee DT, Chan KP, Lee S, Yip PS. Burning charcoal: a novel and contagious method of suicide in Asia. Arch Gen Psychiatry. 2002;59(3):293–4. 10.1001/archpsyc.59.3.293. 11879176 10.1001/archpsyc.59.3.293

[8] Law CK, Yip PS, Caine ED. The contribution of charcoal burning to the rise and decline of suicides in Hong Kong from 1997–2007. Soc Psychiatry Psychiatr Epidemiol. 2011;46(9):797–803. 10.1007/s00127-010-0250-y. 20574845 10.1007/s00127-010-0250-y

[9] Chang SS, Gunnell D, Wheeler BW, Yip P, Sterne JA. The evolution of the epidemic of charcoal-burning suicide in Taiwan: a spatial and temporal analysis. PLoS Med. 2010;7(1):e1000212. 10.1371/journal.pmed.1000212. 20052273 10.1371/journal.pmed.1000212 PMC2794367

[10] Lin JJ, Chang SS, Lu TH. The leading methods of suicide in Taiwan, 2002–2008. BMC Public Health. 2010;10:480. 10.1186/1471-2458-10-480. 20704758 10.1186/1471-2458-10-480 PMC2927545

[11] Chen YY, Yip PS, Chan CH, Fu KW, Chang SS, Lee WJ, Gunnell D. The impact of a celebrity’s suicide on the introduction and establishment of a new method of suicide in South Korea. Arch Suicide Res. 2014;18(2):221–6. 10.1080/13811118.2013.824840. 24620837 10.1080/13811118.2013.824840

[12] Choi YR, Cha ES, Chang SS, Khang YH, Lee WJ. Suicide from carbon monoxide poisoning in South Korea: 2006–2012. J Affect Disord. 2014;167:322–5. 10.1016/j.jad.2014.06.026. 25016488 10.1016/j.jad.2014.06.026

[13] Lyness JR, Crane J. Carbon monoxide poisoning from disposable charcoal barbeques. Am J Forensic Med Pathol. 2011;32(3):251–4. 10.1097/PAF.0b013e3181d03ce7. 20139755 10.1097/PAF.0b013e3181d03ce7

[14] Brooks-Lim EW, Sadler DW. Suicide by burning barbecue charcoal: three case reports. Med Sci Law. 2009;49(4):301–6. 10.1258/rsmmsl.49.4.301. 20025107 10.1258/rsmmsl.49.4.301

[15] Nielsen PR, Gheorghe A, Lynnerup N. Forensic aspects of carbon monoxide poisoning by charcoal burning in Denmark, 2008–2012: an autopsy based study. Forensic Sci Med Pathol. 2014;10(3):390–4. 10.1007/s12024-014-9574-3. 25002407 10.1007/s12024-014-9574-3

[16] Hartwig S, Tsokos M. [Suicidal and accidental carbon monoxide poisonings due to charcoal fires in closed spaces]. Arch Kriminol. 2008;222(1–2):1–13. 18780716

[17] Bolechała F, Strona M. [An unusual case of suicidal carbon monoxide poisoning committed using a portable barbecue grill]. Arch Med Sadowej Kryminol. 2013;63(1):15–20. 23879014

[18] Gunnell D, Coope C, Fearn V, Wells C, Chang SS, Hawton K, Kapur N. Suicide by gases in England and Wales 2001–2011: evidence of the emergence of new methods of suicide. J Affect Disord. 2015;170:190–5. 10.1016/j.jad.2014.08.055. 25254616 10.1016/j.jad.2014.08.055

[19] Chen YY, Bennewith O, Hawton K, Simkin S, Cooper J, Kapur N, Gunnell D. Suicide by burning barbecue charcoal in England. J Public Health (Oxf). 2013;35(2):223–7. 10.1093/pubmed/fds095. 23179241 10.1093/pubmed/fds095

[20] Wirthl I, Schulz R, Schmeling A. [Suicide by means of a charcoal grill. Casuistic report with review of the literature]. Arch Kriminol. 2008;221(5–6):129–37. 18663875

[21] Sinyor M, Williams M, Vincent M, Schaffer A, Yip PSF, Gunnell D. Suicide deaths by gas inhalation in Toronto: an observational study of emerging methods of suicide. J Affect Disord. 2019;243:226–31. 10.1016/j.jad.2018.09.017. 30248633 10.1016/j.jad.2018.09.017

[22] R Core Team. R: A language and environment for statistical computing. https://www.R-project.org (2022). Accessed.

[23] ICECI Coordination and Maintenance Group. International classification of external causes of injuries version 1.2. Consumer Safety Institite. Amsterdam and AIHW National Injury Surveillance Unit, Adelaide; 2004.

[24] Australian Institute of Health and Welfare. Suicide & self-harm monitoring. https://www.aihw.gov.au/suicide-self-harm-monitoring (2023). Accessed 20 May 2023.

[25] Schmitt MW, Williams TL, Woodard KR, Harruff RC. Trends in suicide by carbon monoxide inhalation in King County, Washington: 1996–2009. J Forensic Sci. 2011;56(3):652–5. 10.1111/j.1556-4029.2010.01688.x. 21291470 10.1111/j.1556-4029.2010.01688.x

[26] Huh GY, Jo GR, Kim KH, Ahn YW, Lee SY. Imitative suicide by burning charcoal in the southeastern region of Korea: the influence of mass media reporting. Leg Med (Tokyo). 2009;11(Suppl 1):S563–4. 10.1016/j.legalmed.2009.01.099. 19269229 10.1016/j.legalmed.2009.01.099

[27] Austin A, Winskog C, van den Heuvel C, Byard RW. Recent trends in suicides utilizing Helium. J Forensic Sci. 2011;56(3):649–51. 10.1111/j.1556-4029.2011.01723.x. 21361949 10.1111/j.1556-4029.2011.01723.x

[28] Byard RW. Changing trends in suicides using Helium or nitrogen - A 15-year study. J Forensic Leg Med. 2018;58:6–8. 10.1016/j.jflm.2018.04.007. 29684846 10.1016/j.jflm.2018.04.007

[29] Stephenson L, Wills S, van den Heuvel C, Humphries M, Byard RW. Increasing use of sodium nitrite in suicides-an emerging trend. Forensic Sci Med Pathol. 2022;18(3):311–8. 10.1007/s12024-022-00471-8. 35334075 10.1007/s12024-022-00471-8 PMC9587107

[30] Stephenson L, Van Den Heuvel C, Humphries M, Nash C, Byard RW. Features of fatal pesticide ingestion in South Australia. Med Sci Law. 2023;258024231197914. 10.1177/00258024231197914. 10.1177/00258024231197914 37661826

[31] Winder C. Carbon monoxide-induced death and toxicity from charcoal briquettes. Med J Aust. 2012;197(6):349–50. 10.5694/mja11.10777. 22994834 10.5694/mja11.10777

[32] Dong-hyuk H. Squid Game. South Korea2021.

[33] Sung Jin L. Beef. United States2023.

[34] Pan YJ, Lee MB. Charcoal burning and maternal filicide-suicide trends in Taiwan: the impact of accessibility of lethal methods. J Formos Med Assoc. 2008;107(10):811–5. 10.1016/s0929-6646(08)60195-3. 18926949 10.1016/S0929-6646(08)60195-3

[35] Hon KL. Dying with parents: an extreme form of child abuse. World J Pediatr. 2011;7(3):266–8. 10.1007/s12519-011-0320-6. 21822993 10.1007/s12519-011-0320-6

[36] Laberke PJ, Bock H, Dittmann V, Hausmann R. Forensic and psychiatric aspects of joint suicide with carbon monoxide. Forensic Sci Med Pathol. 2011;7(4):341–3. 10.1007/s12024-011-9224-y. 21327571 10.1007/s12024-011-9224-y

[37] Lee DT, Chan KP, Yip PS. Charcoal burning is also popular for suicide pacts made on the internet. BMJ. 2005;330(7491):602. 10.1136/bmj.330.7491.602-b. 15761009 10.1136/bmj.330.7491.602-b PMC554074

[38] Kinoshita H, Türkan H, Vucinic S, Naqvi S, Bedair R, Rezaee R, Tsatsakis A. Carbon monoxide poisoning. Toxicol Rep. 2020;7:169–73. 10.1016/j.toxrep.2020.01.005. 32015960 10.1016/j.toxrep.2020.01.005 PMC6992844

[39] Veiraiah A. Carbon monoxide poisoning. Medicine. 2020;48(3):197–8. 10.1016/j.mpmed.2019.12.013.

[40] Byard RW. The relationship between chronic disease and drugs/toxins-how important is negative disease-drug synergy? Forensic Sci Med Pathol. 2023. 10.1007/s12024-023-00608-3. 36971894 10.1007/s12024-023-00608-3

[41] Saukko P, Knight B. Knight’s Forensic Pathology. 4th ed. Boca Raton: CRC; 2016.

[42] Ku CH, Huang WH, Hsu CW, Chen YC, Hou YC, Wang IK, et al. Incidence rate and predictors of Globus Pallidus Necrosis after Charcoal burning suicide. Int J Environ Res Public Health. 2019;16(22). 10.3390/ijerph16224426. 10.3390/ijerph16224426 PMC6888208 31718107

[43] Yarid NA, Harruff RC. Globus Pallidus Necrosis Unrelated to Carbon Monoxide Poisoning: retrospective analysis of 27 cases of basal ganglia necrosis. J Forensic Sci. 2015;60(6):1484–7. 10.1111/1556-4029.12838. 26258901 10.1111/1556-4029.12838

[44] Patel F. Carbon copy deaths: carbon monoxide gas chamber. J Forensic Leg Med. 2008;15(6):398–401. 10.1016/j.jflm.2008.01.004. 18586213 10.1016/j.jflm.2008.01.004

[45] Pan YJ, Loi MX, Lan YH, Chen CL, Cheng IC. Perceptions towards charcoal-burning suicide and the surge of this lethal method in Taiwan. PLoS ONE. 2022;17(1):e0262384. 10.1371/journal.pone.0262384. 35061796 10.1371/journal.pone.0262384 PMC8782296

[46] Tsai CW, Gunnell D, Chou YH, Kuo CJ, Lee MB, Chen YY. Why do people choose charcoal burning as a method of suicide? An interview based study of survivors in Taiwan. J Affect Disord. 2011;131(1–3):402–7. 10.1016/j.jad.2010.12.013. 21236495 10.1016/j.jad.2010.12.013

[47] Yip PS, Law CK, Fu KW, Law YW, Wong PW, Xu Y. Restricting the means of suicide by charcoal burning. Br J Psychiatry. 2010;196(3):241–2. 10.1192/bjp.bp.109.065185. 20194548 10.1192/bjp.bp.109.065185

[48] Chang SS, Lin CY, Hsu CY, Chen YY, Yip PSF. Assessing the effect of restricting access to barbecue charcoal for suicide prevention in New Taipei City, Taiwan: A controlled interrupted time series analysis. J Affect Disord. 2021;282:795–802. 10.1016/j.jad.2020.12.147. 33601720 10.1016/j.jad.2020.12.147

