# Supplementary material for: Recent trends in an uncommon method of carbon monoxide suicide
Source: Forensic Sci Med Pathol. 2024 Apr 11;20(3):863–71. doi: 10.1007/s12024-024-00810-x (PMC11525260; doi:10.1007/s12024-024-00810-x)
Supplement: Supplementary file 1 — Supplementary Material 1 [file 12024_2024_810_MOESM1_ESM.docx]

**Appendix A**

**Table A.1 Prevalence and demographic profile of CB suicides reported in the literature**

| **Continent** | **Reference** | **Country(s)** | **Years** | **Proportion(s) reported** | **Demographic profile** |
| --- | --- | --- | --- | --- | --- |
| Asia | Chung & Leung (2001). *Psychiatr Serv* **52**, 836-7.(Chung, Leung 2001) | Hong Kong | 1998-1999 | 3.2% of all suicides used CB | Mean age = 39 years  Most males (64%)  Ethnicity not reported |
|  | Cheng et al. (2017). *PLoS One* **12**, e0175580.(Cheng et al. 2017) | Hong Kong | 1998-2005 | 19.8% of all suicides used CB | Demographic information not reported |
|  | Pan et al. (2010). *J Affect Disord* **120**, 254-7.(Pan et al. 2010) | Taiwan | 1995-2006 | 33.5% of all suicides **in 2006** used CB | Mean age = 38.76 years  Mostly males (74.5%)  Ethnicity not reported |
|  | Chang et al. (2010). *PLoS Med* **7**, e1000212(Chang et al. 2010) | Taiwan | 1991-2007 | 12.9% of all suicides used CB | Rates were higher among males  Age and ethnicity were not reported |
|  | Yip et al. (2012). *Inj Prev* **18**, 187-92.(Yip et al. 2012) | Taipei, Taiwan | 2004-2006 | 30.9% of all suicides used CB | 73.3% males  46.9% aged 40-59 years  Ethnicity not reported |
|  | Law et al. (2011). *Soc Psychiatry Psychiatr Epidemiol* **46**, 797-803.(Law et al. 2011) | Hong Kong | 1997-2007 | 18.3% of all suicides used CB | Not reported |
|  | Law & Leung (2012). *BMC Public Health* **12**, 505.(Law, Leung 2012) | Hong Kong | 2001-2008 | 20.2% of all suicides used CB | 68.2% males  Age and ethnicity not reported |
|  | Lee et al. (2014). *Psychriatry Res* **219**, 518-24.(Lee et al. 2014) | Korea | 2007-2011 | 4.3% of all suicides used CB | Mean age = 40.2 years  83.6% males  Ethnicity not reported |
|  | Chang et al. (2019). *J Affect Disord* **257**, 390-5.(Chang et al. 2019) | Hong Kong | 2002-2013 | 22.6% of all suicides used CB | Mostly 30-49 years of age (>58%)  Mostly male (>65%)  Ethnicity not reported |
|  | Lu et al. (2016). *Congent Medicine* **3**, 1137131.(Lu et al. 2016) | Wuhan and Shanghai, China | 2005-2014 | 82.3% of all non-fire related CO suicides used CB | 52.9% were males  83.2% were aged 18-60 years |
|  | Li et al. (2015). *Forensic Sci Int* **253**, 112-8.(Li et al. 2015) | Wuhan, China | 2009-2014 | 66.7% of CO suicides used CB | Not reported |
|  | Chen et al. (2019). *Iran J Public Health* **48**, 458-64.(Chen et al. 2019) | Sichuan, China | 2008-2016 | 68% of non-fire related CO suicides used CB | More than half male (53.6%)  Mostly adults aged 18-60 years (80.2%)  Ethnicity not reported |
| Europe | Janík et al. (2017). J *Forensic Leg Med* **48**, 23-9.(Janík et al. 2017) | Hradec Králové, Czech Republic | 1947-2006 | 65.9% of CO suicides used CB | 61.6% of CO suicides were male  Mean age (all CO suicides) = 45.1 years  Ethnicity not reported |
|  | Chen et al. (2013). *J Public Health (Oxf)* **35**, 223-7.(Chen et al. 2013) | England | 2005-2007 | 1.9% of all suicides used CB | Mean age = 33.4 years  72.7% males  Ethnicity not reported |
|  | Lyness & Crane (2011). *Am J Forensic Med Pathol* **32**, 251-4.(Lyness, Crane 2011) | Northern Ireland | 2006-2008 | 17.4% of all CO deaths used CB (4/23)* | 3 females, 1 male  Mean age = 44.5 years  Ethnicity not reported |
|  | Gunnell et al. (2015). *J Affect Disord* **170**, 190-5.(Gunnell et al. 2015) | England and Wales, UK | 2001-2011 | <1% of all suicides used CB  ~1-3% of gas suicides used CB | Mean age = 41.1 years  Mostly males (87%)  Ethnicity not reported |
|  | Nielsen et al. (2014). *Forensic Sci Med Pathol* **10**, 390-4.(Nielsen et al. 2014) | Denmark | 2008-2012 | 19 cases, 11 suicides | Mostly males (63.2%)  Middle-aged (38.2 years)  Mostly Caucasian (89.5%), two Nigerian |
|  | Stoll et al. (2017). *Int J Legal Med* **131**, 123-9.(Stoll et al. 2017) | Munich, Germany | 2011-2013 | 18% of all CO deaths used CB* | Mostly males (>75%)  Age and ethnicity not reported |
| North America | Schmitt et al. (2011) *J Forensic Sci* **56**, 652-5.(Schmitt et al. 2011) | Washington, US | 1996-2009 | 6% of all suicides used CO  16% of CO suicides used CB | Most aged 40-59 years (62%)  Mostly males (85%)  Mostly white (88%) |
|  | Sinyor et al. (2019). *J Affect Disord* **243**, 226-31.(Sinyor et al. 2019) | Toronto, Canada | 1998-2015 | 0.9% of all suicides used CB  19% of gas suicides used CB | Mean age = 46 years  Mostly males (86.5%)  Mostly Asian (92.8%) |
| CO = carbon monoxide  CB = charcoal burning  *All intents | | | | | |

**Table A.2 Polynomial regression model output predicting the effect of year on the number of VE suicides**

Call:

lm(formula = Freq ~ Year + I(Year^2), data = dtab[dtab$Method == "VE", ])

Residuals:

| Min | 1Q | Median | 3Q | Max |
| --- | --- | --- | --- | --- |
| -6.2786 | -2.9049 | 0.1286 | 2.3082 | 7.8986 |

Coefficients:

|  | Estimate | Std. Error | t value | Pr(>\|t\|) |  |
| --- | --- | --- | --- | --- | --- |
| (Intercept) | 2.897e+05 | 1.331e+05 | 2.17 | 0.0439 | * |
| Year | -2.864e+02 | 1.325e+02 | -2.162 | 0.0451 | * |
| I(Year^2) | 7.080e-02 | 3.296e-02 | 2.148 | 0.0464 | * |

---

Signif. codes: 0 ‘***’ 0.001 ‘**’ 0.01 ‘*’ 0.05 ‘.’ 0.1 ‘ ’ 1

Residual standard error: 4.367 on 17 degrees of freedom

Multiple R-squared: 0.8819, Adjusted R-squared: 0.868

F-statistic: 63.47 on 2 and 17 DF, p-value: 1.301e-08

*Table A.1 shows a significant quadratic relationship between the number of VE suicides and the year. The rate of change between years is calculated by taking the derivative of this equation, producing the equation:*

$$Rate of change = 0.142 \times Year - 286.4$$

**Figure A.1 Residuals of the polynomial regression model describing the effect of year on the number of VE suicides**


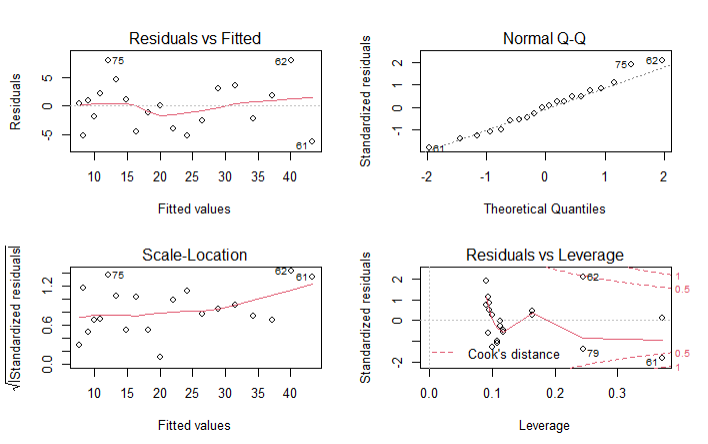


*The residual plots for Table A.1 show that the linearity, normality and homoscedasticity assumptions are met.*

**Table A.3 Poisson regression model output predicting the effect of year on the number of CB suicides**

Call:

glm(formula = Freq ~ Year, family = "poisson", data = dtab[dtab$Method == "CB", ])

Deviance Residuals:

| Min | 1Q | Median | 3Q | Max |
| --- | --- | --- | --- | --- |
| -1.3143 | -0.7334 | -0.5101 | 0.2001 | 1.7029 |

Coefficients:

|  | Estimate | Std. Error | z value | Pr(>\|z\|) |  | |
| --- | --- | --- | --- | --- | --- | --- |
| (Intercept) | -363.22018 | 108.40722 | -3.351 | 0.000807 | *** |  |
| Year | 0.18045 | 0.05381 | 3.353 | 0.000798 | *** |  |

---

Signif. codes: 0 ‘***’ 0.001 ‘**’ 0.01 ‘*’ 0.05 ‘.’ 0.1 ‘ ’ 1

(Dispersion parameter for poisson family taken to be 1)

| Null deviance: | 29.793 on 19 degrees of freedom |
| --- | --- |
| Residual deviance: | 15.033 on 18 degrees of freedom |
| AIC: 43.132 |  |

Number of Fisher Scoring iterations: 5

**Table A.4 Two-sample t-test predicting the relationship between age and method of CO suicide**

data: d.Age by d.Method

t = -2.3912, df = 445, p-value = 0.01721

alternative hypothesis: true difference in means between group CB and group VE is not equal to 0

95 percent confidence interval:

-15.800495 -1.544494

sample estimates:

mean in group CB mean in group VE

37.16667 45.83916

**Table A.5 Two-sample t-test predicting the relationship between COHb concentration and CO suicide method**

data: COHb_Conc by Method

t = -1.1519, df = 435, p-value = 0.25

alternative hypothesis: true difference in means between group CB and group VE is not equal to 0

95 percent confidence interval:

-12.613107 3.291539

sample estimates:

mean in group CB mean in group VE

69.70588 74.36667
